# Semimembranosus Tendon‐Sparing Arthroscopic Inside‐Out Repair of a Medial Meniscus Bucket‐Handle Tear

**DOI:** 10.1002/atn2.70208

**Published:** 2026-08-04

**Authors:** Chengjian Wu, Chengjie Lian, Zhengru Wu, Zhi Chen, Hao Qin, Aiguo Zhou, Hua Zhang

**Affiliations:** ^1^ Department of Orthopaedics Sports Injury Division Fujian Medical University Union Hospital Fuzhou China; ^2^ Department of Orthopedics Chongqing Municipal Health Commission Key Laboratory of Musculoskeletal Regeneration and Translational Medicine The First Affiliated Hospital of Chongqing Medical University Chongqing China

## Abstract

Bucket‐handle tears of the medial meniscus are a common knee injury. Meniscal repair restores stability, reduces degenerative changes, and improves clinical outcomes. Repair may be performed using all‐inside, outside‐in, or inside‐out techniques. Among these, the inside‐out technique is widely regarded as the gold standard because of its reliability and broad applicability. However, blind needle passage during inside‐out repair carries a risk of semimembranosus tendon entrapment. The purpose of this technical note is to describe a step‐by‐step surgical technique for inside‐out repair of medial meniscus bucket‐handle tears that recognizes and addresses entrapment of the semimembranosus tendon.

## Graphical abstract

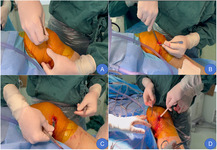

**VIDEO 1:** Semimembranosus tendon‐sparing arthroscopic inside‐out repair of a medial meniscus bucket‐handle tear. The patient is positioned supine with the left leg secured in a leg holder. A posteromedial safety incision is first established, and a retractor is inserted to protect the neurovascular structures. Standard anterolateral and anteromedial arthroscopic portals are then created. Visualization is obtained through the anterolateral portal, and instrumentation is introduced through the anteromedial portal. Arthroscopic examination shows a medial meniscal bucket‐handle tear, which is repaired using the inside‐out technique. Following meniscal repair, the posteromedial safety incision is explored to identify any repair sutures passing through the semimembranosus tendon. Sutures found to penetrate the tendon are carefully retrieved from the deep surface of the tendon and repositioned to prevent postoperative semimembranosus tendon entrapment. The video highlights a practical technique for detecting and preventing this rare complication of inside‐out meniscal repair. Video content can be viewed at https://doi.org/10.1002/atn2.70208.

Meniscal injuries are among the most common lesions of the knee joint, with an annual incidence of 60 to 70 per 100,000 individuals^1^ and an even higher rate among athletes.^2^ Bucket‐handle tears account for 10% to 16% of all meniscal injuries.^3^, ^4^ Compared with partial or total meniscectomy, meniscal repair is associated with a higher reoperation and failure rate;^5^, ^6^ however, repair provides superior chondroprotective effects and reduces the risk of knee osteoarthritis.^7^ Arthroscopic meniscal repair techniques can be categorized into 3 types: inside‐out, outside‐in, and all‐inside.^8^ Among these, the inside‐out technique is considered the gold standard because of its versatility and cost‐effectiveness.^9^ Although some studies have reported comparable clinical outcomes and complication rates between all‐inside and inside‐out repairs, the level of evidence remains low and inconsistent,^10^ and further comparative studies are warranted. Therefore, the inside‐out technique remains the preferred option for bucket‐handle tears. Nevertheless, suture‐related complications may occur with this technique.^11^ Blind needle passage through the posteromedial capsule may inadvertently penetrate the semimembranosus tendon, potentially resulting in postoperative posteromedial pain and restricted knee motion. To date, this complication has been described only in isolated case reports.^12^ The purpose of this technical note is to describe a step‐by‐step surgical technique for inside‐out repair of medial meniscus bucket‐handle tears that recognizes and addresses entrapment of the semimembranosus tendon.

## SURGICAL TECHNIQUE

Patients with severe comorbidities, advanced osteoarthritis (Kellgren‐Lawrence 3 or 4), or asymptomatic chronic bucket‐handle tears are not suitable candidates for repair.^13^ Except for these contraindications, most medial meniscus bucket‐handle tears should be repaired surgically. When ipsilateral malalignment is present, concomitant corrective osteotomy should be considered.

### Patient Positioning and Anesthesia

The patient is placed supine under general or epidural anesthesia. Bilateral knee examinations are performed after anesthesia to assess ligamentous stability, taking care to avoid exacerbation of the meniscal tear. A tourniquet is not routinely used. A lateral thigh post is positioned to facilitate valgus stress during medial compartment exposure.

### Inside‐Out Meniscal Repair

A detailed illustration of this procedure is provided in Video 1. The key technical pearls and pitfalls are summarized in Table 1, and the step‐by‐step operative process is outlined in Table 2.

**TABLE 1:** Pearls and Pitfalls of Arthroscopic Inside‐Out Repair of a Medial Meniscus Bucket‐Handle Tear Without Semimembranosus Tendon

Pearls
• The pie‐crusting technique of the medial collateral ligament can effectively enlarge the medial joint space and improve visualization
• When establishing the medial safe portal, meticulous identification and protection of the sartorius, gracilis, semitendinosus, and saphenous nerve are essential
• During blunt dissection between the medial head of the gastrocnemius and the joint capsule, the dissection should be maintained proximal to the semimembranosus tendon to minimize the risk of neurovascular injury
• The assistant should prepare the needle holder under adequate illumination to promptly retrieve the needle and avoid suture entanglement
• Before knot tying, it is critical to explore whether entrapment of the semimembranosus tendon is present; if necessary, the sutures should be adjusted accordingly
• Use a precise 3‐sided cortical osteotomy to create an osteoperiosteal flap with a preserved distal hinge. This “living hinge” facilitates safe compartment widening while maintaining bone block viability

Pitfalls
• Excessive suture tension or overly tight knots may lead to meniscal ischemia or restricted knee motion
• Suture inadvertently passing through the semimembranosus tendon can cause persistent postoperative pain and limited knee flexion
• Improper suture management can lead to entangled suture ends and prolong operative time
• Before knot tying, it is necessary to check whether the suture has passed through the semimembranosus tendon. If it has, the suture should be retrieved and positioned between the joint capsule and the semimembranosus tendon before securing the knot

**TABLE 2:** Advantages and Disadvantages of Arthroscopic Inside‐Out Repair of a Medial Meniscus Bucket‐Handle Tear Without Semimembranosus Tendon

Advantages
• Inside‐out technique provides strong and stable fixation for bucket‐handle tears
• Smaller needle diameter than all‐inside devices allows more sutures and fixation points with less meniscal trauma
• Allows precise suture placement along the meniscus body and peripheral rim
• Cost‐effective and widely applicable
• Prevents semimembranosus tendon entrapment, reducing postoperative pain

Disadvantages
• Requires an additional medial incision
• Prolongs operative time

After routine prepping and draping, diagnostic arthroscopy is performed through a standard anterolateral portal to evaluate the meniscus and cruciate ligaments. An anteromedial portal is then established under spinal needle guidance. If medial compartment exposure is limited, pie‐crusting release of the medial collateral ligament may be performed.^14^ Granulation tissue along both edges of the tear is debrided, and the meniscal rim is freshened to enhance healing potential. The displaced bucket‐handle fragment is then reduced anatomically using a probe.

A medial safety incision approximately 3 cm in length is made posterior to the superficial medial collateral ligament. Because needles typically exit the capsule with a downward trajectory, one‐third of the incision is placed above and two‐thirds below the joint line. The knee is flexed to 90°. Subcutaneous dissection exposes the sartorial fascia, which is incised to allow retraction of the sartorius, gracilis, and semitendinosus tendons, thereby protecting the saphenous nerve. The interval between the medial head of the gastrocnemius and the joint capsule is bluntly developed, and a retractor is placed to protect neurovascular structures while permitting safe needle exit. Maintaining knee flexion relaxes the hamstrings and gastrocnemius, facilitating needle retrieval.

Commercially available instruments (Double Arm Meniscus Repair Needles, Delta Medical, China) are used for suturing. The arthroscope is maintained in the anteromedial portal, with the anterolateral portal serving as the working portal. Under direct visualization, the cannula is directed toward the tear, and the needle tip is visualized entering the meniscus or capsule before penetration. After passage through the posterior capsule, the knee is flexed to 70° to 90° to facilitate needle retrieval. A second needle is then passed to complete the stitch. Sutures are placed sequentially from posterior to anterior at approximately 5 mm intervals using vertical, oblique, or horizontal configurations depending on tear morphology. Vertical sutures are preferred when feasible because they better capture circumferential meniscal fibers. Needles are retrieved through the safety incision under direct visualization. Once delivered, needle tips are cut, and sutures are tagged without immediate knot tying.

### Exploration and Release of the Semimembranosus Tendon

**FIGURE 1:**
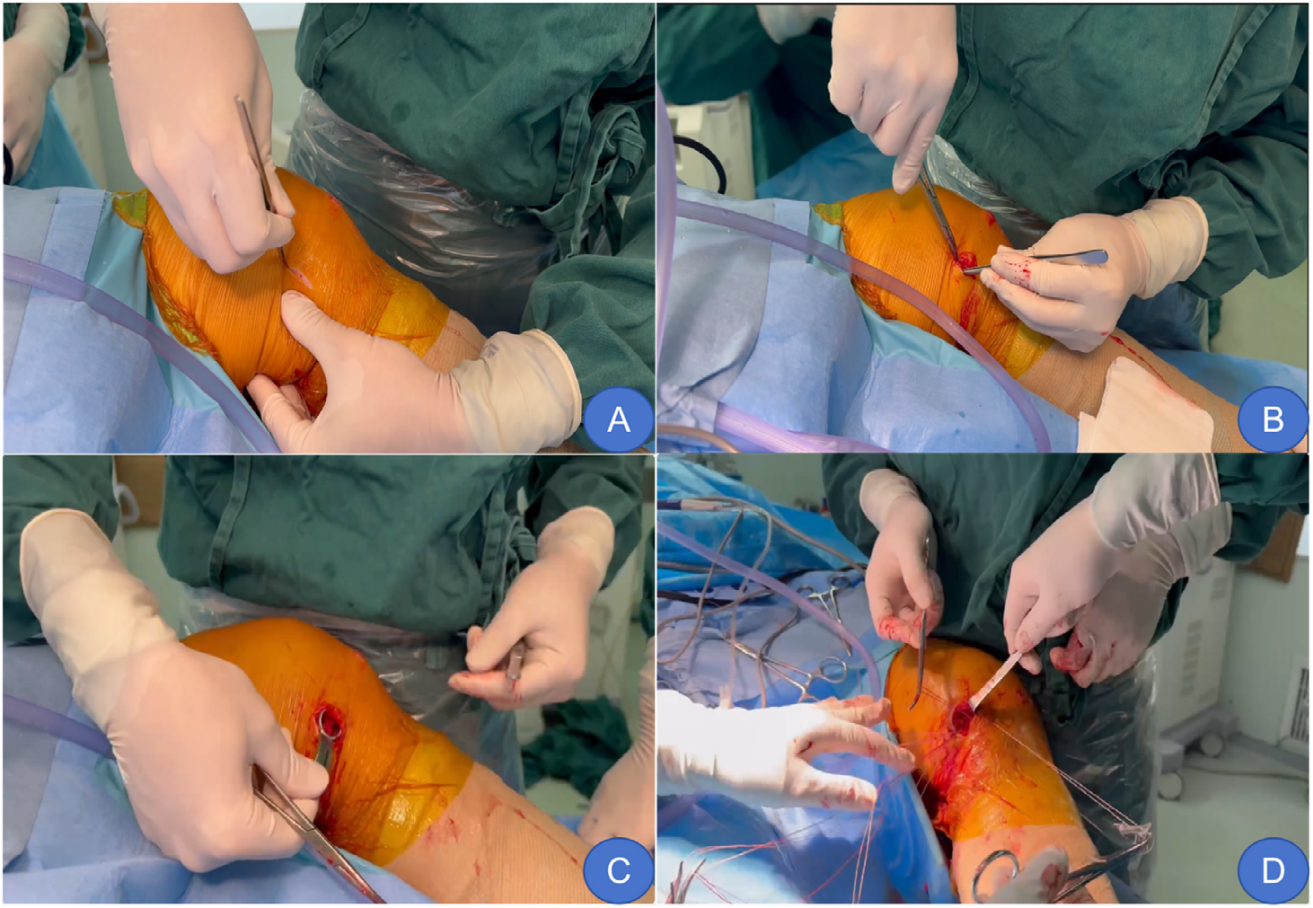
The patient is in the supine position, with the surgical site being the left knee joint. (A) The medial safe incision of the left knee joint. (B) Layer‐by‐layer exposure to the surface of the medial joint capsule of the knee. (C) Insertion of a popliteal retractor into the medial side of the joint capsule to protect the blood vessels and nerves. (D) After completing the inside‐out suturing of the medial meniscus, the risk from the skin incision is guided out through the safe incision.

**FIGURE 2:**
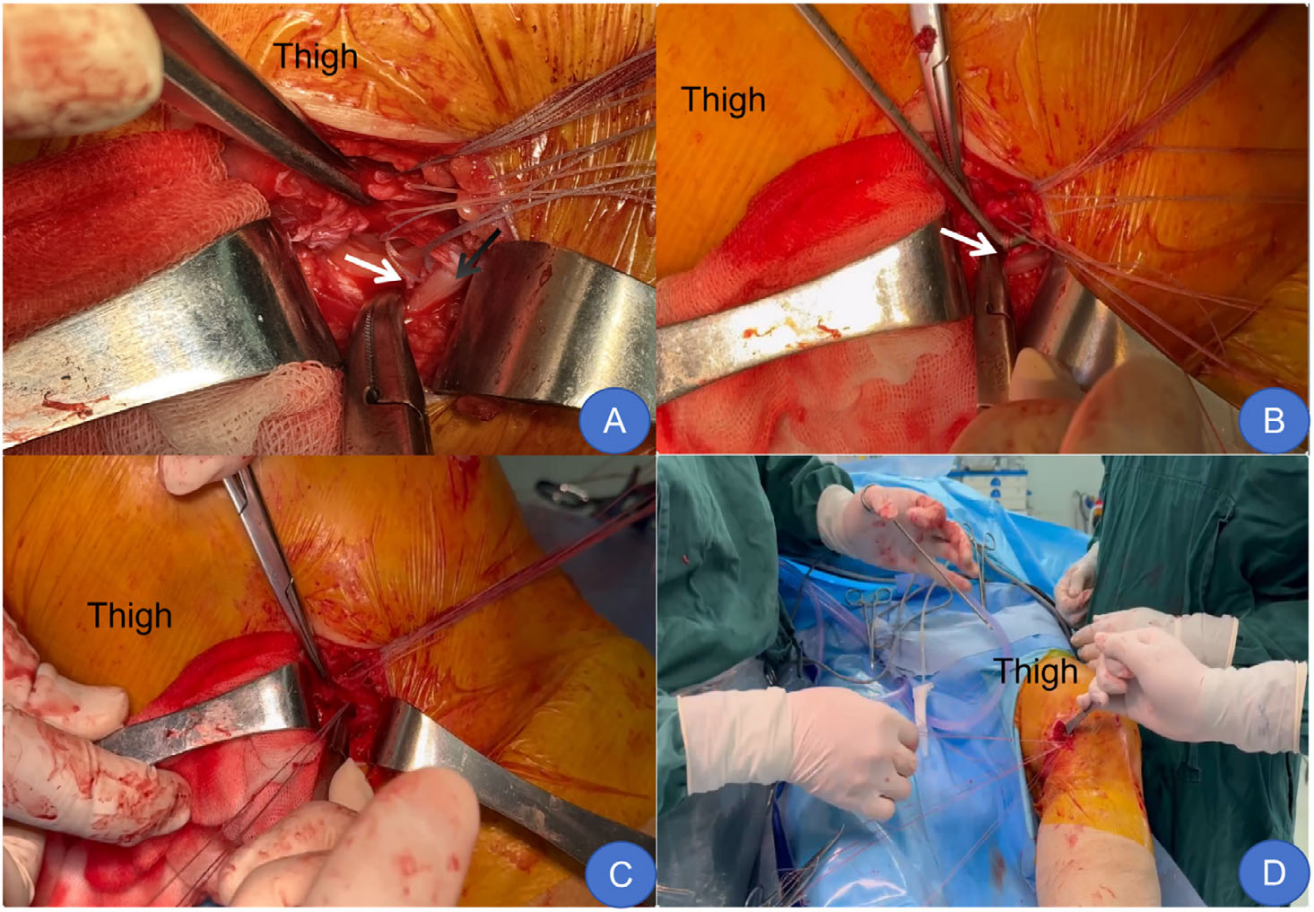
The patient is positioned supine with the surgical site at the left knee joint. (A) Intraoperative exploration reveals the suture passing through the semimembranosus tendon. (B) A probing hook is used to retract the suture from the semimembranosus tendon, thereby alleviating suture entrapment. (C) Re‐exploration confirms that all sutures traversing the semimembranosus tendon have been successfully retrieved, with no signs of entrapment. (D) The sutures are then tied and secured to the medial joint capsule, effectively stabilizing the meniscus.

**FIGURE 3:**
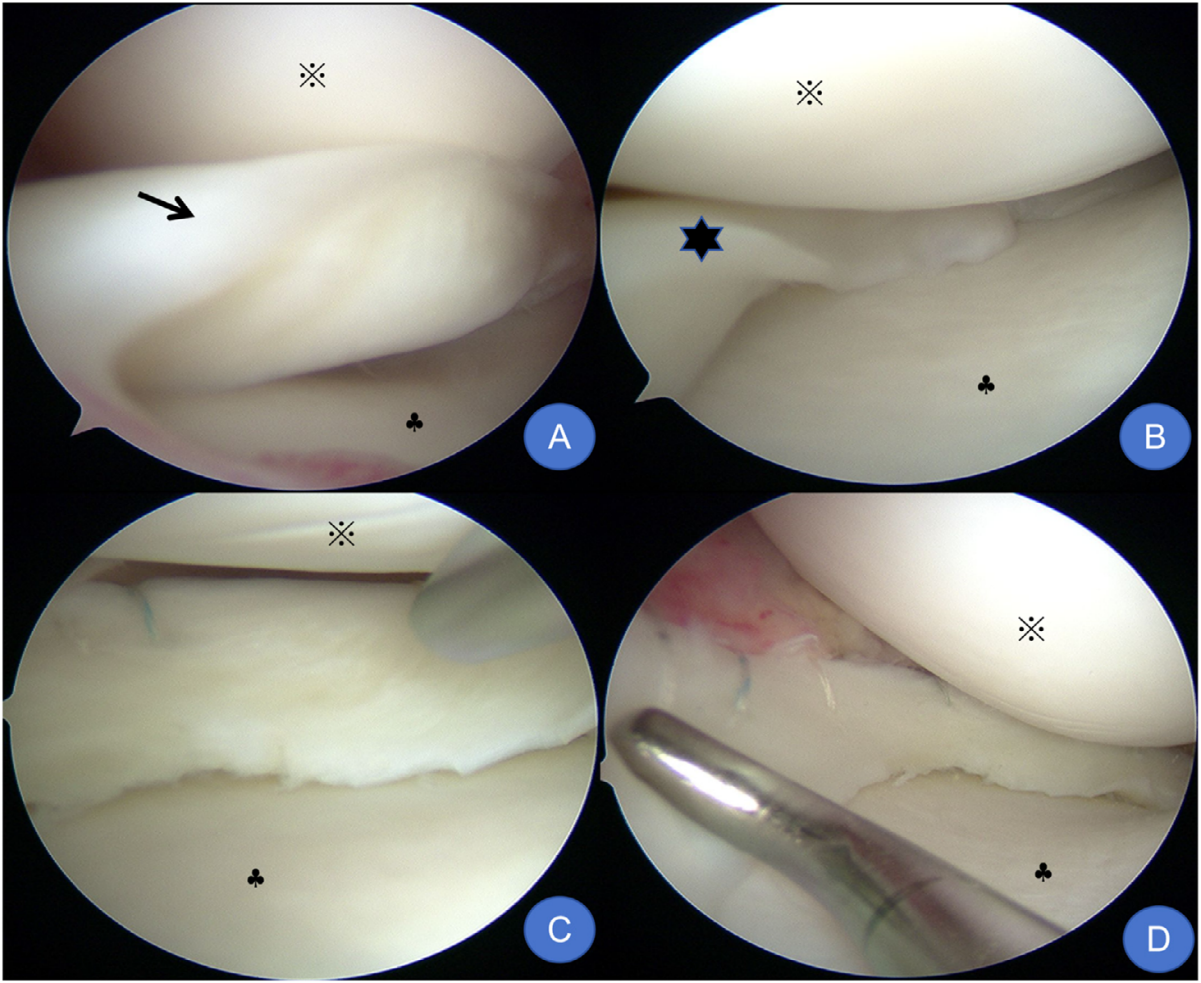
The patient is in the supine position. The arthroscope is introduced into the left knee joint through the anteromedial portal. (A) Exploration reveals a bucket‐handle tear of the medial meniscus. (B) The bucket‐handle tear of the medial meniscus has been reduced. (C) The arthroscope is positioned in the anteromedial portal, and suture instruments are introduced through the anterolateral portal for inside‐out meniscal suturing. (D) Postsuturing, the medial meniscus is successfully reduced, with satisfactory alignment and good stability of the meniscus. (※, medial femoral condyle; ♣, medial tibial plateau; black arrow, bucket‐handle tear of the medial meniscus; star, reduced medial meniscus).

**FIGURE 4:**
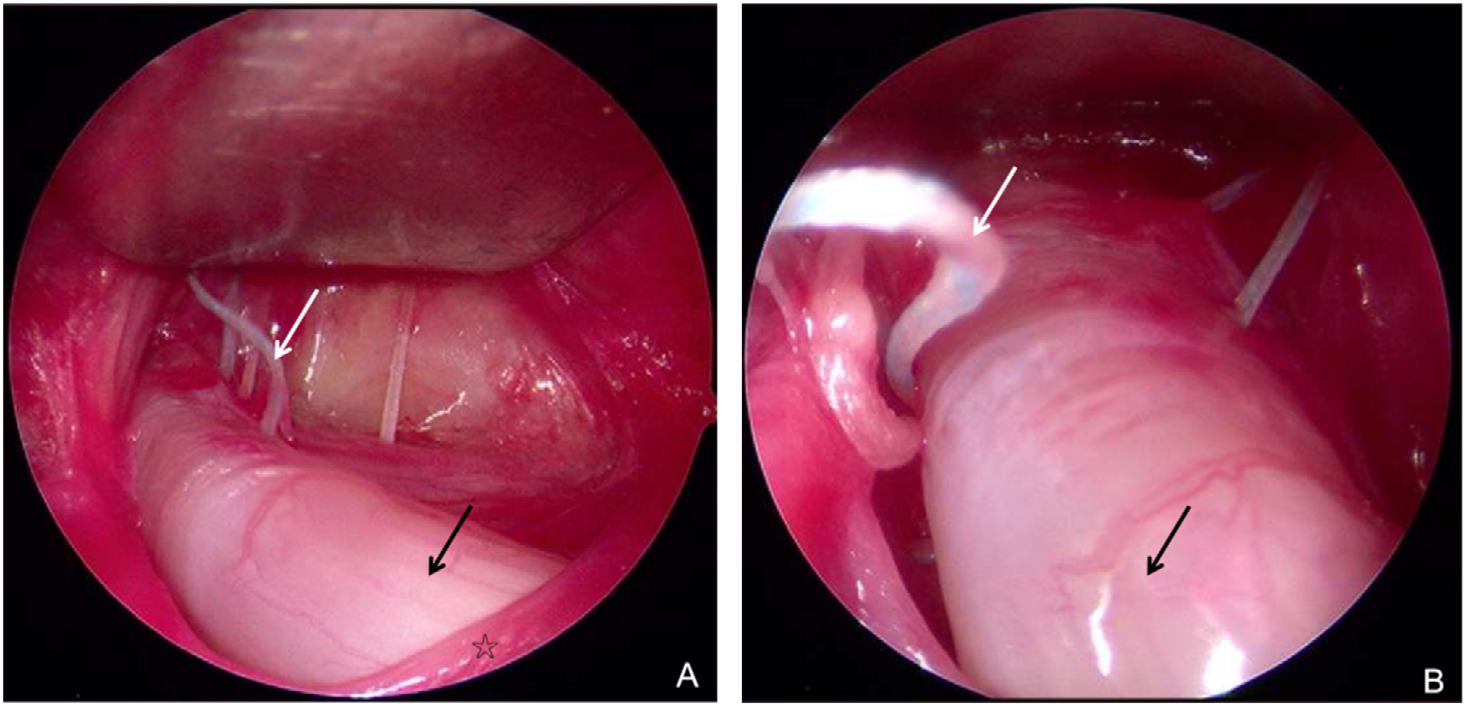
Illustration of the suture retrieval process from the semimembranosus tendon in the left knee. The patient is positioned supine, and the operative field is viewed through the posteromedial safety incision. (A) A meniscal repair suture is shown passing through the semimembranosus tendon. (B) Successful suture retrieval is confirmed, and the semimembranosus tendon is verified to be free of entrapment. (White arrow, suture; black arrow, semimembranosus tendon; asterisk, joint capsule).

The arthroscope is advanced into the posteromedial safety incision to evaluate for inadvertent penetration of the semimembranosus tendon. Sutures that traverse the tendon are identified and carefully released using a probe and then repositioned extracapsularly. Careful suture management is essential to prevent entanglement. Knots are tied sequentially with attention to avoiding overtightening. The joint is thoroughly irrigated, and wounds are closed in a standard layered fashion (Figures 1, 2, 3, 4).

### Postoperative Management

For the first 2 weeks postoperatively, patients are permitted toe‐touch weight‐bearing with the knee immobilized in full extension using a brace, while passive range of motion is allowed up to 45°. During postoperative weeks 3 to 4, partial weight‐bearing is permitted, and knee flexion may progress to 90°. Full weight‐bearing and unrestricted range of motion are allowed only after 8 to 12 weeks postoperatively.^15^

## DISCUSSION

This technical note describes an inside‐out repair technique for medial meniscus bucket‐handle tears with specific measures to prevent semimembranosus tendon entrapment. Previously reported complications of the inside‐out repair include saphenous nerve irritation and popliteal vessel injury.^11^, ^16^ In 2022, Haikal et al.^12^ first reported a case of semimembranosus tendon entrapment, although their patient had undergone repair with all‐inside devices. That patient required revision surgery at 3 months postoperatively for suture removal, which successfully relieved the entrapment and associated pain. In the present technique, we employed a double‐needle inside‐out repair for medial meniscus bucket‐handle tears and intraoperatively identified a similar risk of semimembranosus entrapment. Preventive measures were undertaken to avoid postoperative symptoms caused by tendon incarceration.

The prognosis of meniscal repair largely depends on the size and location of the tear.^17^ In patients undergoing concomitant anterior cruciate ligament reconstruction, reported healing rates reach 87% in the peripheral third and 59% in the middle third zones.^18^ Increasing emphasis on meniscal preservation has led to a decline in partial meniscectomy and a rise in meniscal repair and transplantation procedures.^19^, ^20^ Advances in arthroscopic instrumentation have further facilitated meniscal repair with less invasiveness and shorter operative times.^21^ Currently available meniscal repair techniques include the all‐inside, outside‐in, and inside‐out approaches. The all‐inside and outside‐in techniques are generally better suited for posterior horn and anterior horn tears, respectively, whereas the inside‐out technique remains most commonly used for peripheral tears 1 to 4 cm in length. Cabarcas et al.^10^ reported comparable functional outcomes, failure rates, and complication profiles between inside‐out and all‐inside repairs. Nevertheless, the inside‐out technique, regarded as the gold standard, provides the most secure fixation for bucket‐handle tear patterns.^9^, ^22^ Despite the increasing emphasis on meniscal preservation, partial meniscectomy remains an appropriate option for irreducible tears, avascular‐zone tears, degenerative tears, or when significant deformation of the torn segment is present.^23^

In summary, we recommend that during inside‐out repair of medial meniscus bucket‐handle tears, the semimembranosus tendon should be carefully explored to confirm the absence of tendon entrapment. This preventive step may help reduce postoperative pain and motion limitation associated with tendon incarceration.

## DISCLOSURES

The authors (C.W., C.L., Z.W., Z.C., H.Q., A.Z., H.Z.) declare that they have no known competing financial interests or personal relationships that could have appeared to influence the work reported in this article.
